# Use of a Mechanical Forearm Holder During Elbow Arthroscopy in Lateral Decubitus Position

**DOI:** 10.1016/j.eats.2024.102991

**Published:** 2024-04-04

**Authors:** Guillaume Herzberg, Larry D. Field

**Affiliations:** a Upper Extremity Orthopedic Department, Clinique Parc Lyon, Lyon, France; b Clinique du Val d’Ouest, Écully, France; c Mississippi Sports Medicine and Orthopaedic Center, Jackson, Mississippi, U.S.A.

## Abstract

Proper patient positioning is a key consideration when performing elbow arthroscopy. The lateral decubitus position for arthroscopic management of a variety of elbow disorders has recently gained popularity among elbow surgeons. There are several advantages of the lateral decubitus position. However, the elbow rests in 90° of flexion with the hand hanging free, and an assistant is required to maintain any adjustment to the elbow’s flexion angle. This article describes how a commercially available low-profile multi-articulated mechanical upper-limb holder may be used as a mechanical forearm holder in conjunction with a classic static arm support to provide stable positioning of the elbow in space during elbow arthroscopy. This technique simply and reproducibly provides an effective means to temporarily or permanently maintain the elbow joint in any desired degree of extension or flexion during elbow arthroscopy without the need for an assistant.

Proper elbow positioning is a key consideration when performing elbow arthroscopy irrespective of whether the surgeon chooses supine, prone, or lateral decubitus positioning of the patient.^1^ Currently, the 2 mostly commonly used positions for elbow arthroscopy are the supine-suspended position with the use of a mechanical arm holder^2^ and the lateral decubitus position with the arm supported using a static arm holder and with the forearm and hand hanging free.^3^

When the patient is positioned in the lateral decubitus position, the arm and elbow are relatively stable on an arm support, and the positioning is user-friendly provided that the arm support is proximal enough under the arm and tourniquet to allow adequate flexion of the elbow.^3^ However, an assistant is commonly required during arthroscopy to hold the forearm in an effort to temporarily maintain the elbow in specific degrees of elbow extension (exploration of the olecranon fossa or posterolateral gutter and olecranon process debridement) or hyperflexion (arthrofibrosis release and capitellar osteochondritis dissecans lesion access). Our purpose is to show how a commercially available multi-articulated mechanical upper-limb holder may be used as a mechanical forearm holder in conjunction with a classic static arm support to provide a simple and secure means to temporarily or permanently maintain the elbow during arthroscopy in any desired extended or flexed position without the need for an assistant.

## Technique

**Fig 1:**
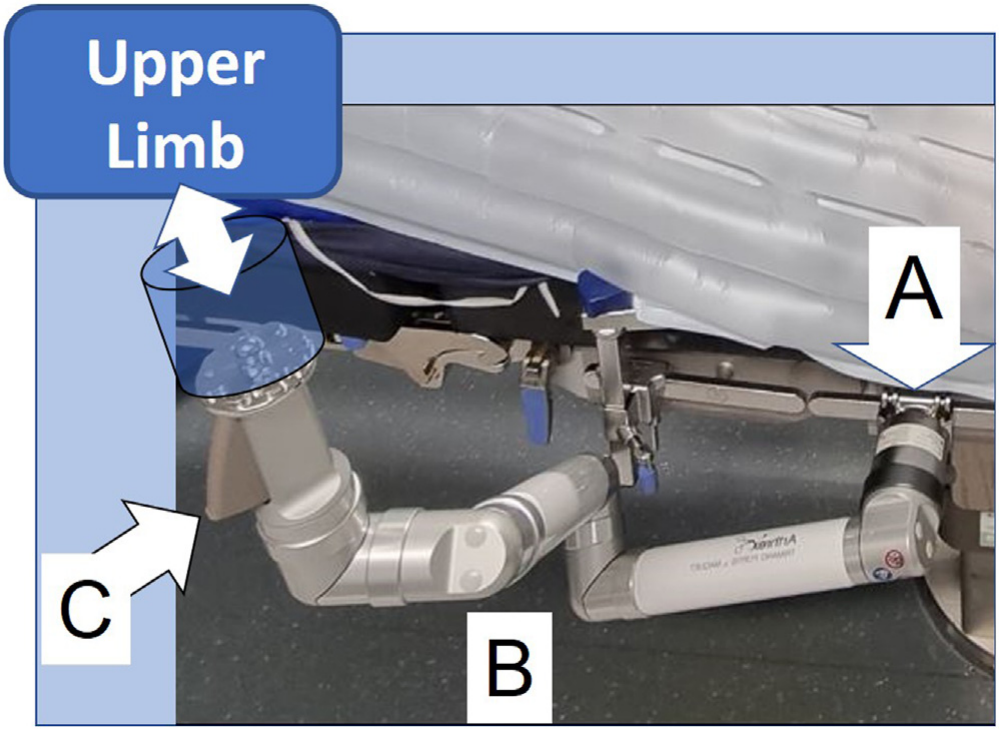
Trimano multi-articulated upper-limb positioner. (A) Easy, handy screw fixation to operating room table rail. (B) Trimano multi-articulated mechanical system. (C) Light-touch handle for effortless adjustment of Trimano device in space.

**Fig 2:**
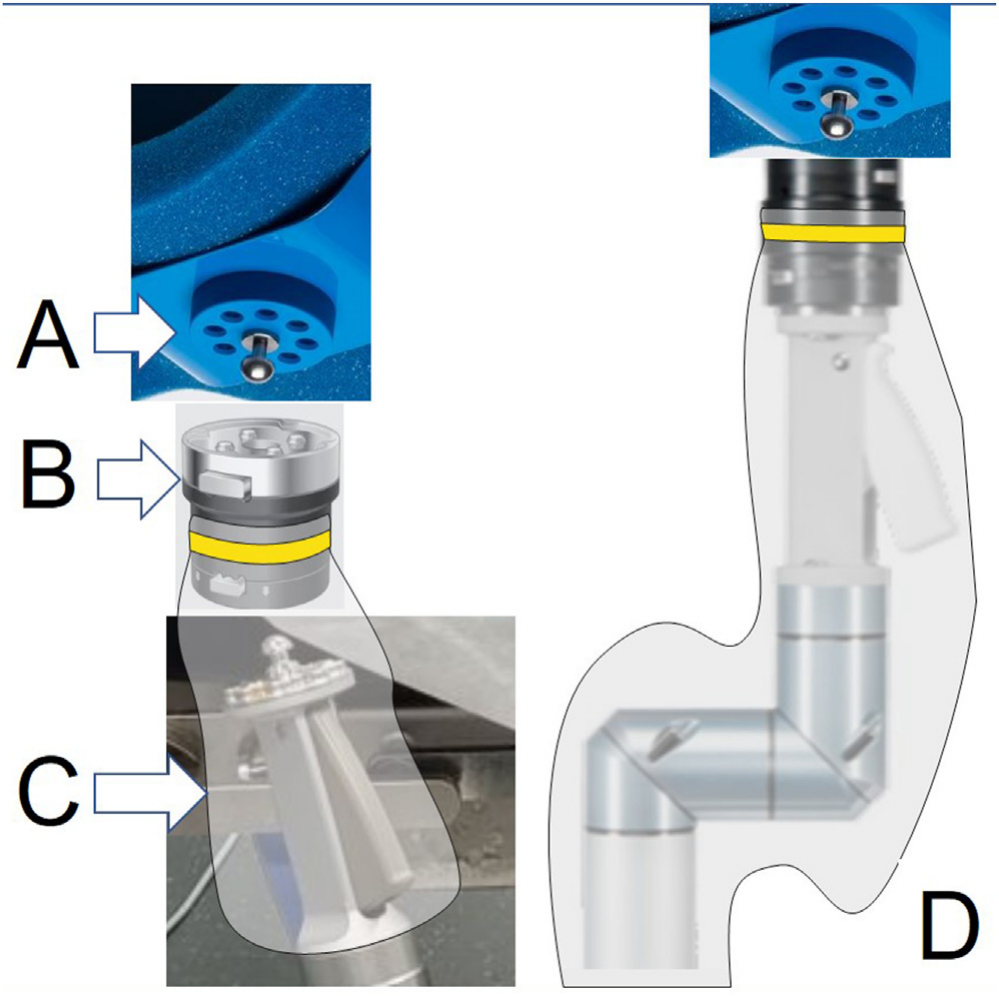
Trimano upper-limb positioner assembly with upper limb. (A) Sterile disposable foam arm or forearm holder. (B) Sterile autoclavable adapter. (C) Sterile transparent arthroscopy sheath. (D) Final construct of link between nonsterile Trimano device and sterile upper limb. The position of the whole Trimano multi-articulated system is controlled by the surgeon through the light-touch handle that is visible and accessible through the transparent sheath.

The Trimano Fortis device (Arthrex, Naples, FL) is a nonsterile upper-extremity positioning, flexible, multi-articulated mechanical system (Trimano articulated upper-limb positioner) that attaches to any operating room table rail on one end and then to the patient’s sterile upper extremity on the other end (Fig 1). The link between the free end pins of the nonsterile Trimano device and the sterile upper extremity is made through 2 devices: a sterile disposable circumferential foam arm or forearm holder and a sterile autoclavable adapter that is stuck to a sterile transparent arthroscopy sheath. The sterile transparent sheath is advanced along the extent of the Trimano mechanical arm so that this device can be brought into the operative field with its position controlled by the surgeon using a light-touch handle that is visible and accessible through the transparent sheath (Fig 2).

**Fig 3:**
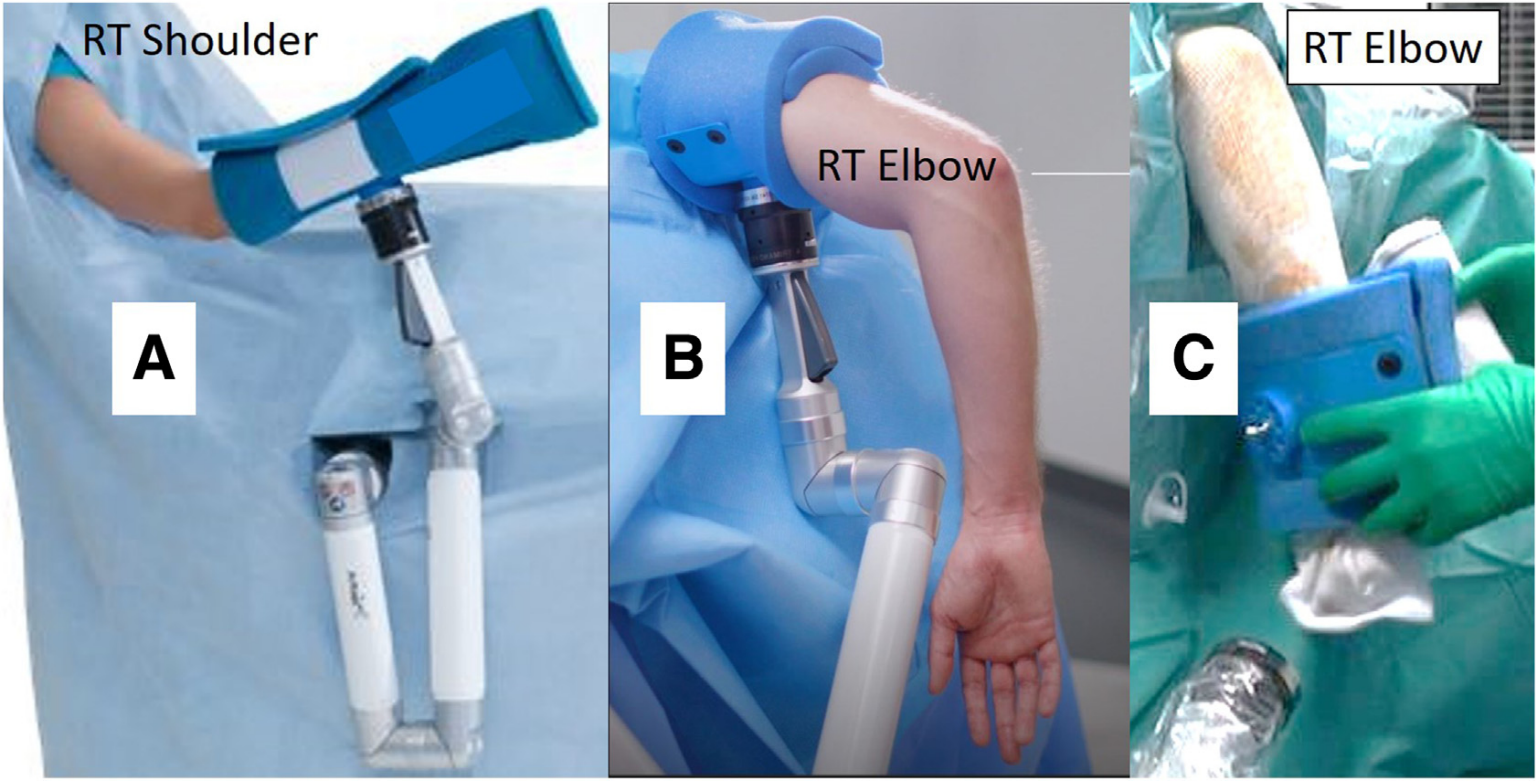
(A) Trimano low-profile upper-limb positioner with disposable sterile foam large forearm holder as used for shoulder surgery. (B) Trimano upper-limb positioner with disposable sterile foam arm holder as commercially available for elbow arthroscopy. (C) Trimano low-profile upper-limb positioner with disposable sterile foam arm around wrist used as forearm holder for elbow arthroscopy as described in article (Video 1). (RT, right.)

The Trimano Fortis device is widely used in shoulder surgery through a connection to the operative extremity via a large sterile forearm holder (Fig 3A). A sterile elbow holder is also available that can be used as an arm holder to replace the classic static arm support during elbow arthroscopy (Fig 3B).

**Fig 4:**
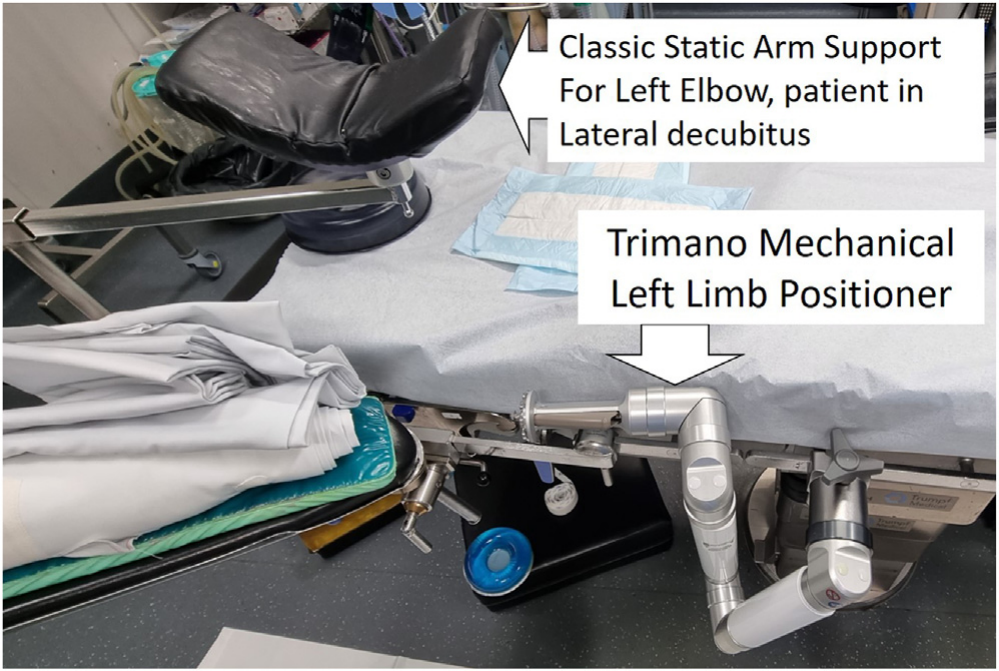
Operating room table nonsterile preparation using classic static arm holder in combination with Trimano low-profile upper-limb positioner for left elbow arthroscopy with patient in lateral decubitus position.

**Fig 5:**
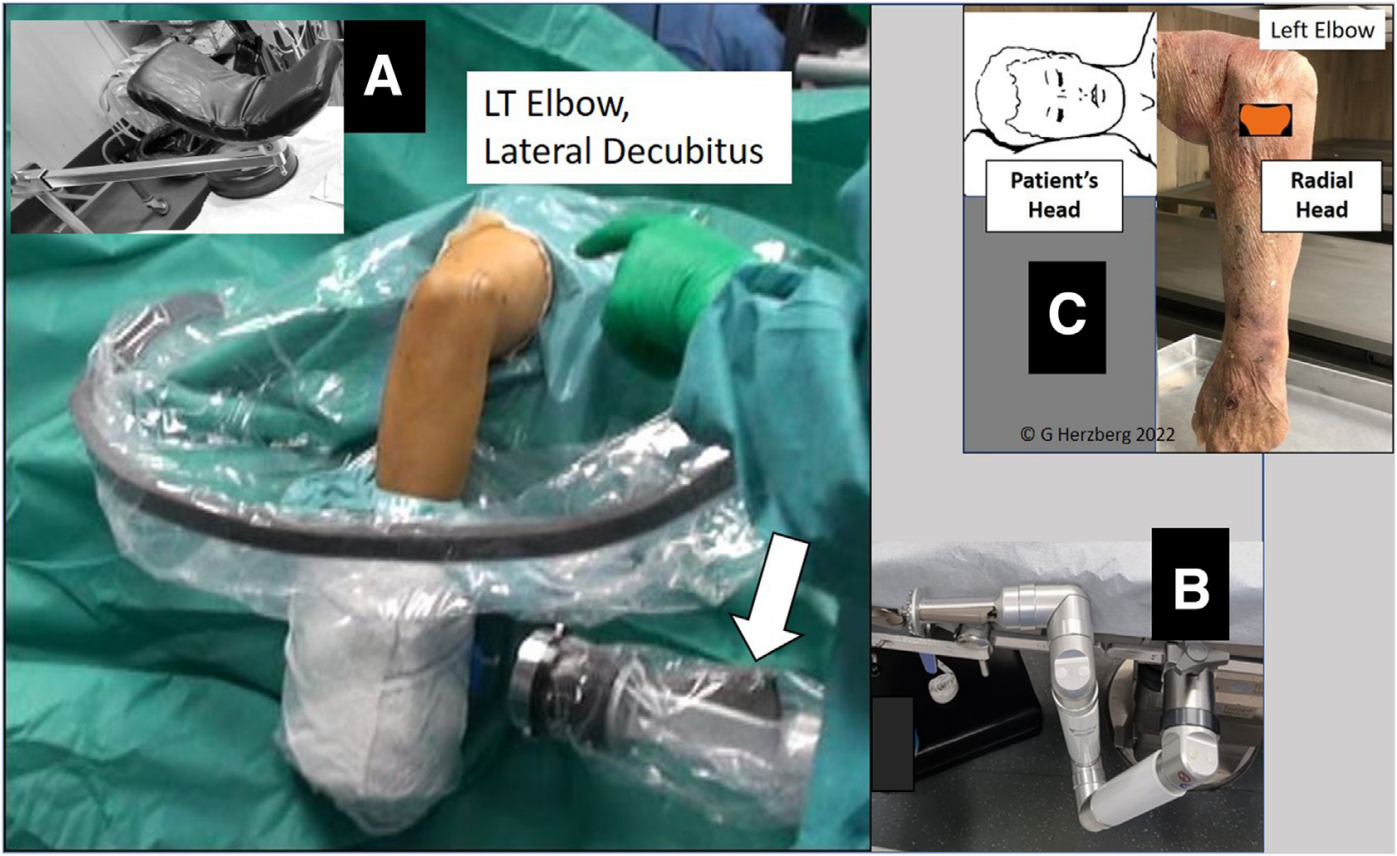
Final operating room table sterile setup for left (LT) elbow arthroscopy, with patient in lateral decubitus position, using combination of classic static arm support (A) and Trimano low-profile upper-limb positioner (B). The white arrow indicates the light-touch handle that the surgeon will see through the sterile transparent sheath and use for placing and stabilizing the elbow in any desired position of flexion or extension. (C) A useful tip for the orientation of the surgical team is that the radial head of the elbow faces the patient’s head in the lateral decubitus position, easily indicating the lateral side of the elbow.

Instead of using the Trimano Fortis device as an arm holder with a disposable sterile foam arm for elbow arthroscopy, our technique is performed using the low-profile Trimano foam arm holder as a forearm holder in combination with a classic static arm support. The commercially available sterile foam arm is placed around the patient’s wrist (Fig 3C). The surgeon can easily control and stabilize the degree of elbow flexion and extension through the light-touch handle without the need for an assistant. This combination of a classic arm support and the low-profile Trimano device to be attached to the forearm (Figs 4 and 5) is very effective in stabilizing the elbow during elbow arthroscopy and has the added benefit of allowing the surgeon to place the elbow in any desired flexion-extension or valgus-varus position without the need for a surgical assistant (Video 1).

Once general anesthesia has been induced, the patient is positioned on a standard operative table in the lateral decubitus position with an axillary roll. The arm equipped with a tourniquet is placed on a classic static arm support (Fig 5A) that is proximal enough to allow free full flexion of the elbow. It is useful for the operating team to remember that the radial head, thus the lateral elbow side, faces the head of the patient when facing the elbow placed on the arm support (Fig 5C).

The body may be stabilized using classic padded supports or a bean bag. The patient is placed leaning slightly forward so that the elbow extends beyond the edge of the operative table. Placing the patient slightly forward facilitates arthroscopic access to the anterior elbow compartment because the elbow can achieve increased flexion in this position.^3^ A pneumatic tourniquet is placed high in the axilla and inflated to 250 mm Hg after limb exsanguination. A Trimano Fortis nonsterile device is fixed to the operative table caudal to the elbow so that its distal end may easily reach the patient’s wrist (Fig 5B).

**Fig 6:**
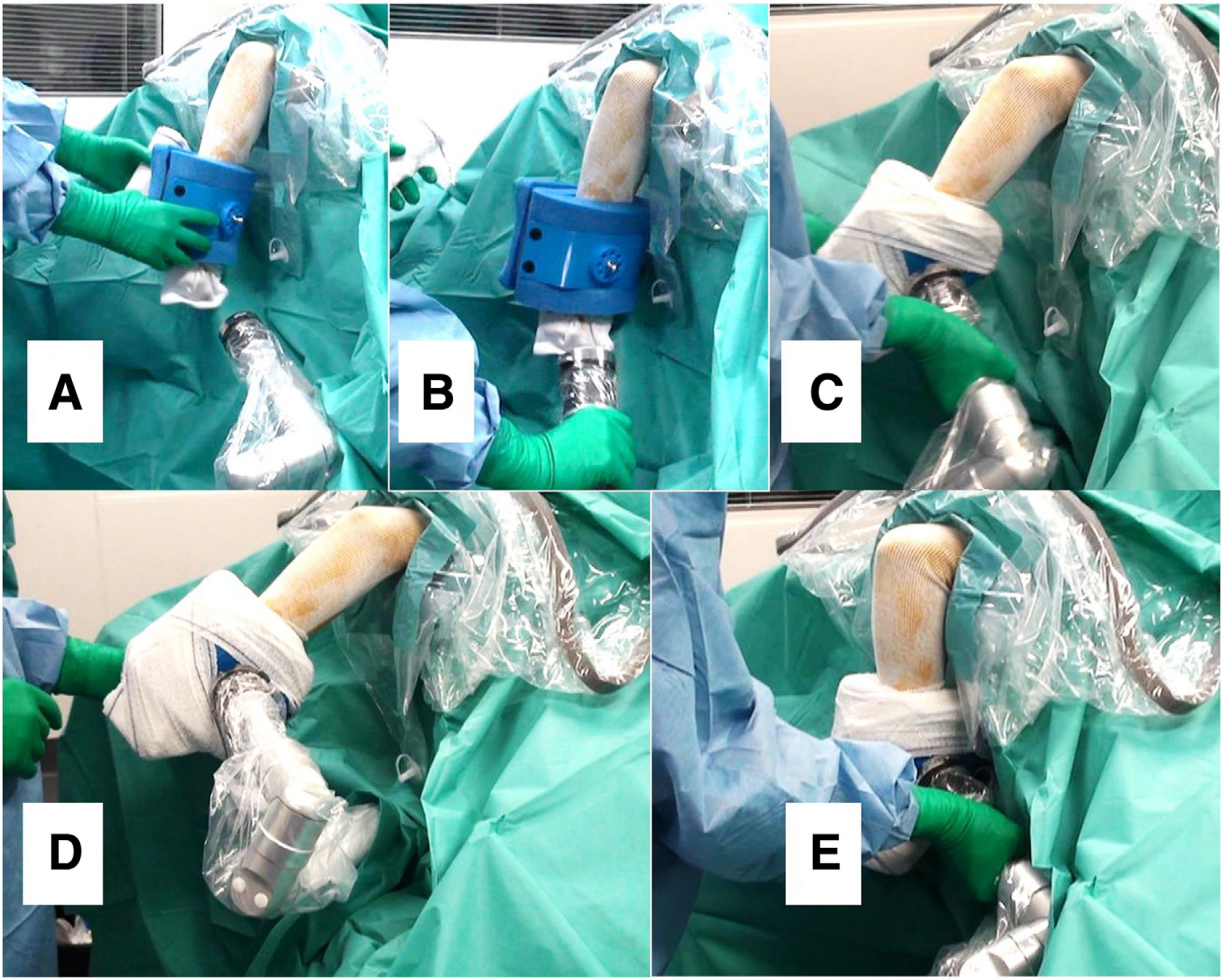
Step-by-step Trimano multi-articulated mechanical upper-limb positioner setup for elbow arthroscopy, in left elbow, with patient in lateral decubitus position and arm supported at tourniquet level by static classic arm support. (A) The sterile foam holder is placed around the wrist. (B) The sterile adapter surrounded with transparent sterile sheath is linked to the foam wrist and forearm holder. (C) The surgeon uses the light-touch Trimano handle through the sterile sheath to place the elbow in the desired position of flexion or extension. (D) Stable 30° flexion position of elbow as provided by Trimano device. (E) Stable 95° flexion position of elbow as provided by Trimano device. As for any elbow arthroscopy, the static arm support must be proximal enough at the arm level to allow full flexion of the elbow.

The patient undergoes sterile preparation and draping, and a transparent, sterile arthroscopy sheath is placed around the entire length of the Trimano Fortis arm (Fig 6). Next, instead of allowing the hand and forearm to hang free using gravity force, a Trimano disposable sterile elbow support is wrapped around the patient’s wrist and secured. The wrist support is then easily connected to the free end of the device’s articulating arm. Once the wrist support and Trimano articulating arm are sterilely connected, the position of the elbow may be readjusted as often as required. In addition, the patient’s forearm can be easily and repeatedly disconnected from the device’s articulating arm as necessary depending on the surgeon’s needs, such as when performing intraoperative elbow stability testing or range-of-motion assessment.^4^^,^^5^ Combining the support that the static arm holder provides with the additional security afforded by the Trimano forearm holder results in a very stable position of the elbow at any flexion or extension angle. Temporarily adding fixed varus or valgus stress to open the joint spaces laterally or medially during elbow instability management can also be incorporated intraoperatively using this device. Moreover, stabilizing the elbow using these combined arm and forearm supports results in the surgeon’s ability to precisely position the elbow joint without the need for an assistant. The limited space immediately adjacent to the elbow during arthroscopy creates challenges if both the surgeon and an assistant are required to remain in very close proximity to the elbow during the arthroscopic procedure. The low-profile Trimano Fortis device usually eliminates the need for an assistant to hold the forearm, thus allowing more room for the surgeon to work. The rigid stability provided by the supplemental use of the Trimano device allows for the maintenance of a motionless elbow position. This ability to hold the elbow motionless can be beneficial as increased elbow flexion allows the surgeon to maximize the space in the anterior compartment. Likewise, the ability to maintain slight extension of the elbow without interruption allows for optimized access to the posterior-compartment working space, as well as the posterolateral and posteromedial gutters. Similarly, if access to the anterior capitellum is desired, a stable temporarily fixed elbow in hyperflexion may help deliver the capitellum from the radial head and will facilitate the creation of an efficient and accurately positioned direct lateral portal.

**Fig 7:**
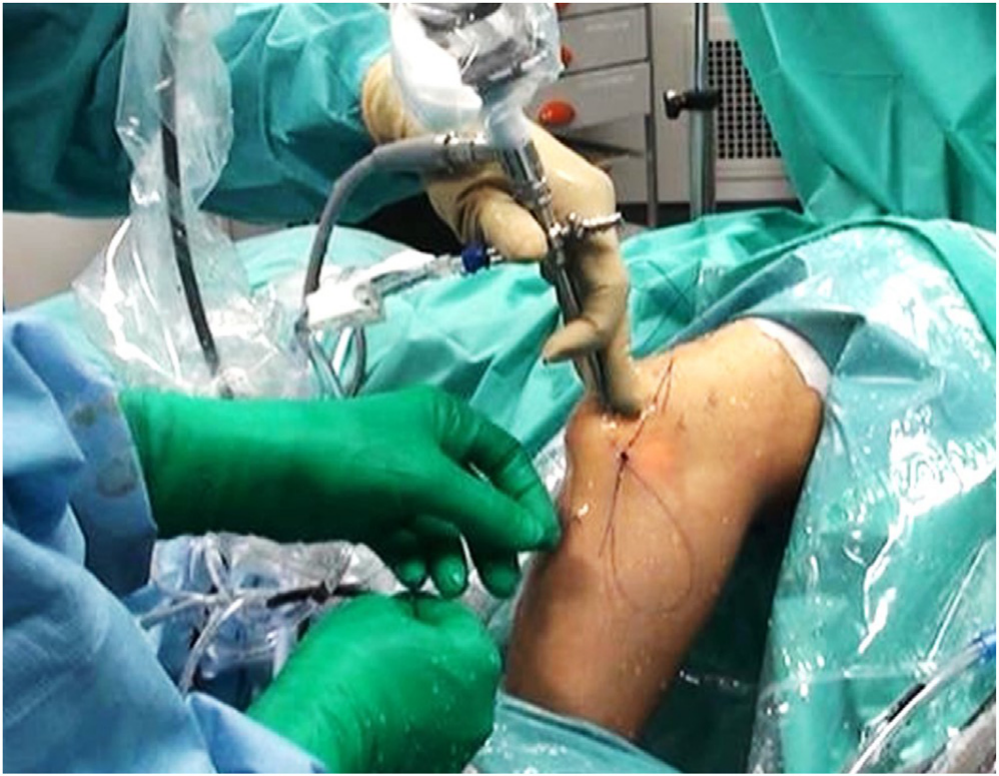
Lateral ulnar collateral ligament plicature performed on left elbow with patient in lateral decubitus position. The elbow is stabilized at 45° of flexion using the low-profile Trimano device as described in the article. There is no need for an assistant to hold the forearm. The assistant can efficiently hold the arthroscope to provide a stable arthroscopic view while the surgeon can safely perform knot tying. The assistant keeps his right hand available to help the surgeon.

We have used the Trimano device as a forearm mechanical holder with lateral decubitus positioning in our last 14 elbow arthroscopies (1 for osteochondritis dissecans, 6 for arthroscopic debridement and loose body removal, 3 for posterolateral rotatory instability, and 4 for arthroscopic lateral epicondylitis release). We have found that the ability to quickly position and securely maintain the elbow in a desired flexed or extended position (or by valgus or varus stress) without the help of an assistant has proved a significant advantage. For example, during arthroscopic imbrication for a posterolateral rotatory instability procedure,^6^ the assistant did not need to maintain the elbow at 60° of flexion during knot tensioning. Instead, he used one hand to maintain a stable camera position into the posterolateral elbow gutter while keeping his other hand free to provide instruments while the surgeon tensioned the knots (Figs 3 and 7). Table 1 further illustrates the advantages and disadvantages of our positioning technique.

**Table 1:** Advantages and Disadvantages of Use of Mechanical Forearm Holder During Elbow Arthroscopy in Lateral Decubitus Position

Technique advantages
The technique eliminates the need for an assistant solely for maintaining elbow positioning in space during elbow arthroscopy.
The technique provides easy, consistent and/or temporary rigidity of upper-extremity positioning during any arthroscopic elbow procedure.
Freedom of movement for the surgeon during elbow arthroscopy is increased owing to the low-profile of the positioning system and the absence of an assistant dedicated to arm or elbow control.
Technique disadvantages
Additional costs are associated with the device purchase and sterile single-use consumables. (A disposable sterile support wrapped around the patient’s wrist—easily locked to or unlocked from the tip of the Trimano device—is required for each procedure).

## Discussion

Proper patient positioning is a key consideration when performing elbow arthroscopy because joint space distraction is not possible.^2^^,^^3^^,^^7^, ^8^, ^9^, ^10^ The surgeon must rely on the distension of the capsule by a continuous water stream to create^4^, ^5^, ^6^^,^^11^, ^12^, ^13^, ^14^, ^15^ and maintain articular elbow “intra-compartmental room” with a consistent view. Inadequate positioning may have a direct negative impact on the ability of the arthroscope to visualize and access specific parts of the elbow compartments.

In 1992, O’Driscoll and Morrey^16^ first described a modification of prone positioning by placing the patient in the lateral decubitus position with the arm secured in an arm holder. Subsequently, the lateral decubitus position for arthroscopic management of a variety of elbow disorders has gained popularity among elbow surgeons. There are several benefits to arthroscopy in the lateral decubitus position, including a relatively stable position of the arm and upper extremity, as well as the ability to freely manipulate the elbow during surgery.^3^ Some surgeons have proposed the use of distraction in the lateral decubitus position to enlarge the articular space.^17^ but this may be difficult to replicate.

When arthroscopy is performed in the lateral decubitus position, a low-profile padded arm holder is traditionally positioned in front of the patient to support the proximal humerus. A commercially available mechanical arm holder kit (Trimano Fortis; Arthrex) may be used as a humeral support instead of a static classic arm support.

However, performing elbow arthroscopy in the lateral decubitus position using only a standard arm holder leaves the forearm and hand hanging free. Consequently, an assistant is required during a portion of most arthroscopic elbow procedures to position and maintain the forearm in varying degrees of flexion or extension depending on the procedure. For example, the assistant is typically required to hold the elbow in extension to facilitate posterior-compartment access to the olecranon fossa and posterolateral gutter. Similarly, an assistant may be necessary to temporarily position the elbow at flexion angles greater than 90° during procedures treating anterior osteochondritis dissecans lesions.^18^ Using the Trimano Fortis elbow kit as a mechanical mobile forearm holder in conjunction with a classic arm holder offers significant advantages during elbow arthroscopy, freeing the assistant to perform other tasks instead of being required to maintain the elbow in any position other than the 90° position afforded by gravity.

## Disclosures

Both authors (G.H., L.D.F.) declare that they have no known competing financial interests or personal relationships that could have appeared to influence the work reported in this paper.

## Supplementary Data

ICMJE author disclosure forms

ICMJE author disclosure forms

Video 1 Use of Trimano Fortis mobile arm in conjunction with static mechanical arm holder and sterile wrist adapter. Several arthroscopic elbow procedures demonstrate the stability, range of elbow positioning, and ease of use of the disclosed patient positioning system.
